# Death of Dr. W. G. Drake

**Published:** 1884-09-20

**Authors:** 


### DEATH OF DR. W. G. DRAKE.

Dr. Drake, a prominent and highly esteemed member of the Profession in Atlanta, died on Thursday, the 11th inst.

At a meeting of the Physicians of the city on the 12th inst, Dr. J. F. Alexander was called to the chair, and Dr. W. S. Elkin appointed Secretary.

On a motion, a committee of three, consisting of Drs. W. D. Bizzell, R. C. Word and E. C. Roach was appointed to draught resolutions expressive of the sentiments of the meeting.

The committee, having retired a few minutes, thus reported:

"Your committee beg to report as follows:

"It is with profound sorrow that we have to chronicle the death of our Professional Brother, Dr. W. G. Drake. Genial, competent and faithful to every public and private trust, he was what every true physician should be, a laborious, honorable and good man.

"We tender our heartfelt sympathies to the family of the deceased.

"We further recommend that the Profession attend the funeral in a body, and that the Secretary furnish a copy of these proceedings to the family of Dr. Drake, to the city papers and to the medical journals of Atlanta."

